# *Lactococcus* strains with psychobiotic properties improve cognitive and mood alterations in aged mice

**DOI:** 10.3389/fnut.2024.1439094

**Published:** 2024-08-01

**Authors:** Kan Gao, Cailing Chen, Zhiyao Zheng, Qiuling Fan, Haifeng Wang, Yanjun Li, Su Chen

**Affiliations:** ^1^Research and Development Department, Hangzhou Wahaha Group Co., Ltd., Hangzhou, China; ^2^Hangzhou Wahaha Technology Co., Ltd., Hangzhou, China; ^3^Key Laboratory of Food and Biological Engineering of Zhejiang Province, Hangzhou, China; ^4^College of Animal Science, MOE Key Laboratory of Molecular Animal Nutrition, Zhejiang University, Hangzhou, China

**Keywords:** *Lactococcus*, gut microbiota-brain axis, cognition, mood, 5-HT

## Abstract

Aging often accompanies cognitive and mood disturbances. Emerging evidence indicates that specific probiotics mitigate cognitive and mood dysfunctions. Strains within *Lactococcus*, a subgroup of probiotics, including *Lactococcus lactis* and *Lactococcus cremoris* are shown beneficial effects on brain functions via the gut microbiota-brain axis (GBA). Our previous study identified two *Lactococcus* strains (*L. lactis* WHH2078 and *L. cremoris* WHH2080) with the ability to promote the secretion of gut 5-hydroxytryptophan (5-HTP), the precursor of the GBA mediator 5-hydroxytryptamine (5-HT). In this study, the modulatory effects of WHH2078 and WHH2080 on cognitive and mood alternations were investigated in aged mice. Oral administration of WHH2078 and WHH2080 (1 × 10^9^ CFU/mL/day) in aged mice (12-month-old) for 12 weeks significantly improved cognitive and depressive-and anxiety-like behaviors. The neuronal loss, the 5-HT metabolism dysfunction, and the neuroinflammation in the hippocampus of aged mice were restored by WHH2078 and WHH2080. the disturbances in the serum tryptophan metabolism in aged mice were unveiled by metabolomics, notably with decreased levels of 5-HT and 5-HTP, and increased levels of kynurenine, 3-hydroxykynurenine, and indolelactic acid, which were reversed by WHH2078 and WHH2080. Regarding the gut microbial community, WHH2078 and WHH2080 restored the increased abundance of *Firmicutes*, *Desulfobacterota*, and *Deferribacterota* and the decreased abundance of *Bacteroidota* and *Actinobacteriota* in aged mice. The beneficial effects of the two strains were linked to the modulation of 5-HT metabolism and gut microbiota. Our findings point to the potential role of *Lactococcus* strains with 5-HTP-promoting abilities as therapeutic approaches for age-related cognitive and mood disorders.

## Introduction

Aging is often associated with cognitive alterations and mood disorders, such as gradual declines in learning and memory abilities, depression, and anxiety (1). The hippocampus is closely involved in the aging process and is well-recognized as one of the important regions in the central nervous system (CNS) in the modulation of age-related cognition and mood functions (2). Age-related cognitive declines and mood disorders are linked to disturbances in the hippocampal levels of 5-hydroxytryptamine (5-HT) and brain-derived neurotrophic factor (BDNF), which exert modulatory function in the synaptic plasticity and neurogenesis (3). Meanwhile, neuroinflammation is known as a common feature during aging and neurodegeneration, with the increased production of proinflammatory cytokines (e.g., interleukin-1beta [IL-1β], IL-6, and tumor necrosis factor-alpha [TNFα]) (4). Cognitive enhancers, such as modafinil (5), or antidepressant drugs, selective serotonin reuptake inhibitors (SSRIs) as an example (6), are increasingly being prescribed, whereas associated unpleasant side effects such as headache, diarrhea, and addiction issues (5, 6). Therefore, it is imperative to improve the quality of life using novel, effective, and safe treatments.

Probiotics are renowned as groups of bacteria that can confer beneficial effects on the hosts, including humans and animals (7). Growing evidence suggests that certain probiotics such as *Lactobacillsus* strains [particularly *Lactiplantibacillus plantarum* (8, 9) and *Lactobacillus helveticus* (10)] and *Bifidobacterium* strain [*Bifidobacterium longum* (11)], can improve cognitive and mood dysfunctions through the gut microbiota-brain axis (GBA) (12), which is the bidirectional interplay between the gut microbes and CNS function (13). As a subgroup of probiotics, *Lactococcus lactis* serves as a classical starter culture in the dairy fermentation industry (14). Recently, strains within *Lactococcus* including *L. lactis* (15) and *Lactococcus cremoris* (16) have also shown their beneficial effects on CNS functions, pointing to the potential for the modulation of age-related cognitive and mood alterations.

Emerging evidence indicates that 5-HT (over 95%) is mainly produced in the gut, and endogenous 5-HT plays a vital modulator along the GBA (17). However, the gut-derived 5-hydroxytryptophan (5-HTP), as the 5-HT precursor, but not 5-HT itself, can act on the CNS directly through the blood–brain barrier (BBB) (17). Therefore, probiotics with the gut 5-HTP-promoting ability may have the potential modulatory effects on brain functions. According to an *in vitro* method for screening potential psychobiotics (18), our previous study identified two *Lactococcus* strains (*L. lactis* WHH2078 and *L. cremoris* WHH2080) with the ability to promote the secretion of gut 5-HTP (15).

Therefore, the present study aimed to explore the beneficial effects of WHH2078 and WHH2080 on age-related cognitive and mood dysfunctions. The alterations in cognitive behaviors, depressive-and anxiety-like behaviors, hippocampal neuronal loss, neuroinflammation, neurochemical factors, circulating tryptophan metabolic profile, as well as gut microbiome composition in aged mice following the administration of WHH2078 and WHH2080, were investigated.

## Materials and methods

### *Lactococcus* strains preparations

*L. lactis* WHH2078 and *L. cremoris* WHH2080 were sourced from the Probiotic Microorganisms Conservation Center in Hangzhou Wahaha Technology Co., Ltd. (Hangzhou, China). The basic information of strains such as the origin, collection time and location, and classification are shown in Supplementary Table S1. *L. lactis* WHH2078 and *L. cremoris* WHH2080 were preserved in the National Microbial Culture Collection Center under the entry numbers CGMCC21831 and GDMCC63219. 16S rRNA sequences of two strains were uploaded onto the NCBI GenBank database under the accession numbers MW805736 and OQ439175.

*L. lactis* WHH2078 and *L. cremoris* WHH2080, which were previously frozen at −80°C, were thawed at room temperature and inoculated into fresh M17 medium (2% v/v, Haibo-Biotech Co., Ltd., Qingdao, China), then cultured anaerobically at 37°C for 16 h. Cells were separated by centrifugation (4,000 *g*, 20 min), then washed in sterile phosphate-buffered saline (PBS), and re-dispersed in an M17 medium adding 25% glycerol at a concentration of 1 × 10^12^ colony-forming unit (CFU) /milliliter (mL). Up until use, aliquots of the resuspended strain cells were kept at −80°C.

### Mouse experiment design

Thirty-two male C57BL/6 mice including eight 2-month-and twenty-four 12-month-old mice were obtained from the SLAC Animal Laboratory Co., Ltd. (Shanghai, China). All mice were housed in a controlled environment with a temperature of 25 ± 0.2°C, humidity of 55 ± 10%, and a 12/12 h light/dark schedule (lights time: 8:00 to 20:00), and had free access to standard chow (Cat No. p1101f-25, SLAC Animal Laboratory) and sterile water at all times. All procedures related to mouse care and handling were carried out in compliance with the Ethics Code Permit WHH2023002, which was approved by the Animal Care and Use Committee of Hangzhou Wahaha Technology Co., Ltd. The experiment was conducted with the least amount of mouse suffering possible.

Mice were randomly assigned to one of four groups after a week of acclimatization (*n* = 8): a control group consisting of 2-month-old mice, an aged group consisting of 12-month-old mice, an aged+WHH2078 or aged+WHH2080 group consisting of 12-month-old mice, respectively. The sample size was predetermined by a prior study (19). Before the animal experiments, the number of strains in the frozen storage was counted. For the daily gavage in the animal experiments, the required working concentration is 1 × 10^9^ CFU/mL, which was achieved by diluting the strains with sterile saline through a gradient dilution process. Mice in the control and aged groups were orally given 200 μL sterile saline per day, and mice in the aged+WHH2078/WHH2080 groups were orally given 200 μL of WHH2078 or WHH2080 suspension (1 × 10^9^ CFU/mL) for 12 consecutive weeks. Behavioral tests were performed from week 9 to week 12. The mice’s body weight changes and health status were monitored weekly during the experiment.

**Figure 1:**
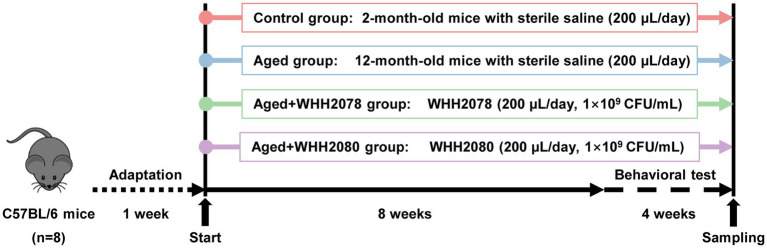
The experimental design. Mice were adapted for 1 week and then treated with *Lactococcus* strain WHH2078, WHH2080, or saline for 12 consecutive weeks (*n* = 8). All mice were subjected to behavioral tests during the last 4 weeks of the experiment.

After all behavioral tests were completed, mice were euthanized by decapitation at week 12. Blood samples were collected, and serum samples were obtained by centrifugation (5,000 *g*, 20 min). Fecal samples and fresh hippocampal and proximal colonic tissues were obtained and stored immediately at −80°C until use. The workflow of the mouse experiment is illustrated in Figure 1.

### Y-maze test

The Y-maze test was conducted according to a method previously described (20). In brief, each mouse was placed in the center of the three-arm maze (40 cm × 8 cm × 12 cm), and the number of entries into each arm was tracked during the 8 min recording time using the JLBehv-YM monitoring system (Jiliang Technology Instruments, Shanghai, China). Spontaneous alternation was defined as entries into three arms in a row. The spontaneous alternation (%) ratio was determined as the numbers of spontaneous alternation/ (total arm entries-2) × 100.

### Shuttle-box test

The shuttle-box test was conducted in this study according to the previous research method (20). The experiment was conducted for four consecutive days, each mouse was placed into a shuttle box (40 cm × 20 cm × 40 cm) equipped with the light and sound stiustimulus as well as 0.2 mM electric shock and received 20 times of stimulation daily. The quantity of active avoidance reactions was recorded by the JLBehv-STG system (Jiliang Technology Instruments).

### Novel object recognition test

The novel object recognition (NOR) test was performed using a method previously reported (20). In brief, the experiment was conducted for five consecutive days, each mouse was put into a plastic box (50 cm × 50 cm × 50 cm) that contained two identical blue wooden cylinders for 10 min each day for 4 days. A red triangular prism was put in place of one of the cylinders on the fifth day. During a 5 min test, the amount of time each mouse spent investigating two objects was recorded using the JLBehv-LAM system (Jiliang Technology Instruments). The exploration time for the novel object divided by the entire exploration time yielded the recognition index (RI) (Time exploring the novel object – time exploring the familiar object) /total exploring time was used to determine the discrimination index (DI).

### Open field test

The open field test (OFT) was conducted following the previously mentioned method (15). Briefly, each mouse was positioned in the middle of the plastic box (50 cm × 50 cm × 50 cm), and the locomotor activity was monitored for 5 min by the JLBehv-LAM system (Jiliang Technology Instruments). The amount of time spent by each mouse in the central area (20 cm × 20 cm) and the distance traveled (cm) were assessed.

### Forced swim test

The forced swim test (FST) was carried out in accordance with a previous method (15). Briefly, each mouse was put in the glass cylinder (30 cm × 20 cm × 50 cm) filled with sterile water (approximately 25 cm deep, 26 ± 2°C) to train swimming for 10 min on the first day. Mice were placed into the cylinder the next day to swim for 5 min. Each mouse’s immobility time during the 5 min test was tracked by the JLBehv-FSM (Jiliang Technology Instruments).

### Nissl staining analysis

The hippocampus Nissl staining study was performed using a previously published technique (20). Hippocampal neurons were counted in the representative areas such as dentate gyrus (DG), cornu ammonis 1 (CA1), and CA3 using the Image-Pro Plus software 6.0 (Media Cybernetics Inc., Silver Springs, MD, United States).

### Cytokines and neurochemical factors determination

The concentrations of key neuroinflammatory cytokines such as IL-1β, IL-6, and TNFα, and neurochemical factors including mature BDNF (mBDNF) and 5-HT in the hippocampus were evaluated using commercial ELISA kits (Jiancheng Taihao Biotechnology Co., Ltd., Nanjing, China) following the standard guidelines. The accuracy and precision of ELISA tests are shown in Supplementary Table S1. Using the previously described ultra-performance liquid chromatography (UPLC) technique (15, 20), the amounts of 5-HTP in the hippocampus and colonic samples were determined.

### Tryptophan metabolism-targeted metabolomics

A total of thirty-one tryptophan metabolites were assessed in this study, the relevant information of each metabolite is provided in Supplementary Table S2. The serum samples from mice for tryptophan metabolism-targeted metabolomics were prepared according to the method previously reported (20). The metabolomics analysis was performed using the SCIEX AnalystWork Station 1.6.3 and Sciex MultiQuant 3.0.3 platform (Biotree Biotech Co., Ltd., Shanghai, China). The final concentrations of each tryptophan metabolite were calculated (21) and used in the comparison of differences among groups.

### High throughput sequencing of 16S rRNA and bioinformatics

The fecal DNA samples for High throughput sequencing of 16S rRNA were prepared according to the method previously described (20). Amplicons were sequenced by the PacBio Sequel system (LC-Bio Technology Co., Ltd., Hangzhou, China). The bioinformatics was conducted using the QIIME 2.0 pipeline (LC-Bio Technology Co., Ltd.). Amplicon sequencing variants (ASVs) were collected and assigned using the SILVA database. The Shannon index was used to estimate microbial alpha diversity, the Bray-Curtis distance-based principal component analysis (PCoA) was used to estimate microbial beta diversity, and permutational analysis of variance (PERMANOVA) was used to assess differences within and between groups. The abundances of the different gut microbial taxa were distinguished using linear discriminant analysis (LDA) effect size (LEfSe) (LDA score ≥ 4.0). After applying false discovery rate (FDR) corrections (22), the q values from the LefSe analysis that were less than 0.05 were deemed statistically significant. The 16S rRNA sequencing data generated in the present study were uploaded onto the NCBI SRA database under the accession number PRJNA1021314.

### Statistical analysis

The medians and 95% confidence intervals (CIs) for all data were displayed. Data were analyzed with R software 4.1. When the data were normally distributed, the one-way analysis of variance (ANOVA) was carried out after performing *post hoc* Turkey’s multiple comparisons. The Kruskal-Wallis test was conducted when the data were not in a normal distribution. When the *p* values were less than 0.05, the differences among/between groups were deemed statistically significant.

## Results

### Effects of *Lactococcus lactis* WHH2078 and *Lactococcus cremoris* WHH2080 on behaviors in aged mice

**Figure 2:**
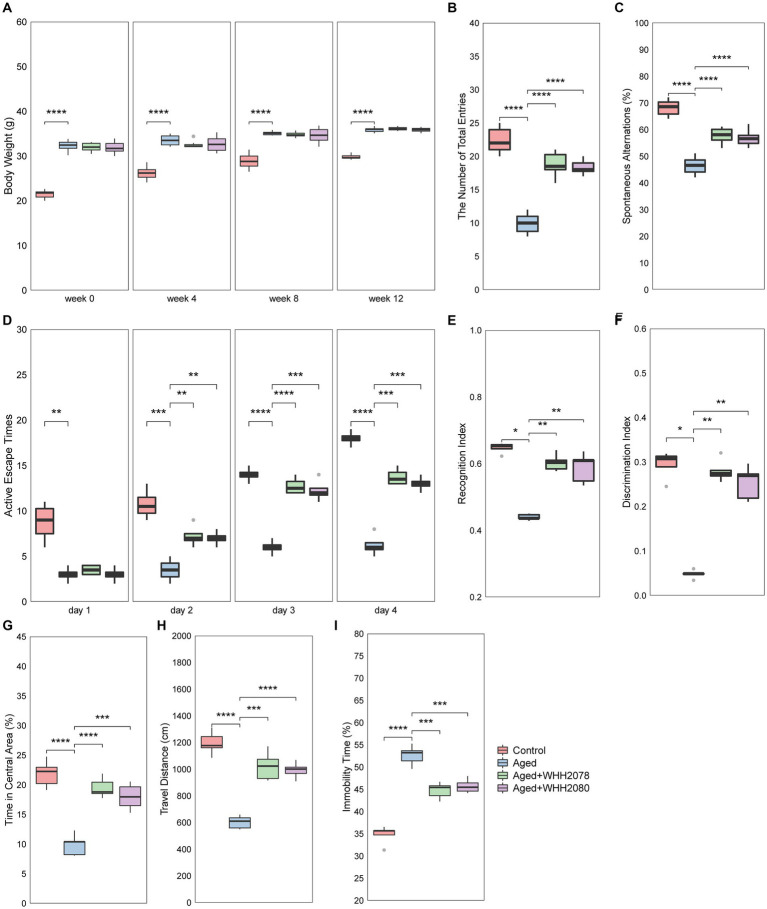
The behavioral alterations in mice. **(A)** Body weight. The number of total arm entries **(B)** and spontaneous alternations **(C)** in the Y-maze test. **(D)** The active escape times in the shuttle-box test. The recognition index **(E)** and discrimination index **(F)** in the novel object recognition (NOR) test. The time in the central area **(G)** and travel distance **(H)** in the open-field test. **(I)** The immobility time in the forced-swim test. Data are presented as medians ±95% CI, *n* = 8. * *p* < 0.05, ** *p* < 0.01, *** *p* < 0.001, **** *p* < 0.0001.

Compared to the control group, the mice in the aged group exhibited higher body weights throughout the experiment (Figure 2A, *p* < 0.0001). The administration of either WHH2078 or WHH2080 did not yield significant effects on the body weights of aged mice (Figure 2A, *p* > 0.05). The learning and memory functions of mice were evaluated through cognitive behavioral tests. In the Y-maze test, when compared to the young mice, the aged mice showed significantly fewer total entries (Figure 2B, *p* < 0.0001) and spontaneous alternations (%) (Figure 2C, *p* < 0.0001). The administration of WHH2078 and WHH2080 significantly reversed the total entries (Figure 2B, *p* < 0.0001) and spontaneous alternations (Figure 2C, *p* < 0.0001) in the aged mice. In the shuttle-box test, the aged mice showed lower active escape responses at all-time points during the experiment period than young mice (Figure 2D, *p* < 0.01). The mice in the WHH2078 and WHH2080 groups displayed significant increases in active escape times on days 2, 3, and 4 (Figure 2D, *p* < 0.01; Figure 2D, *p* < 0.001; Figure 2D, *p* < 0.001), respectively. In the NOR test, significant decreases in RI (Figure 2E, *p* < 0.05) and DI (Figure 2F, *p* < 0.05) were observed in the aged group, which were increased by treatment with WHH2078 and WHH2080 (Figures 2E,F, *p* < 0.01). The tracking movement in the NOR test can be seen in Supplementary Figure S1.

The depressive-and anxiety-like behaviors were also assessed. The aged mice in the OFT test showed significant reductions in the amount of time spent in the central area (Figure 2G, *p* < 0.0001) and the travel distance (Figure 2H, *p* < 0.0001) as compared to the young mice, which were restored by the administration of WHH2078 and WHH2080 (Figures 2G,H, *p* < 0.001). The OFT tracking movement can be seen in Supplementary Figure S2. In the FST test, a significantly increased immobility time was observed in the aged mice (Figure 2I, *p* < 0.0001), and the immobility time was significantly reversed by the treatment of WHH2078 and WHH2080 (Figure 2I, *p* < 0.001).

### Effects of *Lactococcus lactis* WHH2078 and *Lactococcus cremoris* WHH2080 on hippocampal neuron, neuroinflammation, and neurochemical factors in aged mice

**Figure 3:**
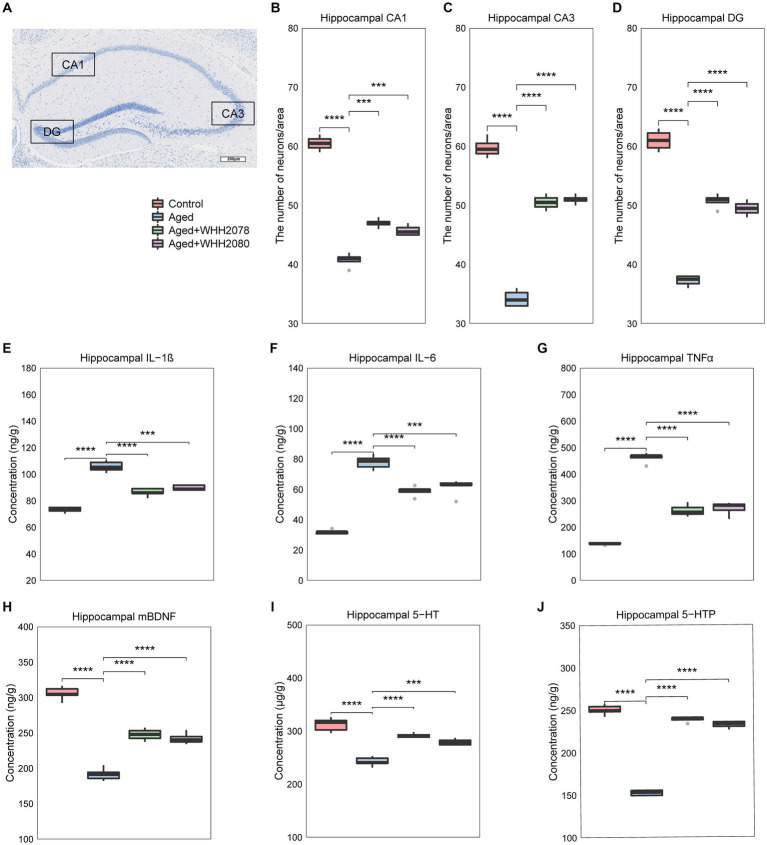
The changes in neuronal loss, neuroinflammation, and the levels of neurochemical parameters in the hippocampus of mice. **(A)** The representative image of Nissl-stained hippocampal CA1, CA3, and DG areas in aged mice. The number of neuronal cells in the CA1 area **(B)**, CA3 area **(C)**, and DG area **(D)**. The levels of hippocampal IL-1β **(E)**, IL-6 **(F)**, and TNFα **(G)**. The levels of hippocampal mature BDNF **(H)**, 5-HT **(I)**, and 5-HTP **(J)**. Data are expressed as medians ±95% CI, *n* = 8. * *p* < 0.05, ** *p* < 0.01, *** *p* < 0.001, **** *p* < 0.0001.

Neuronal quantification was performed in designated subregions of the hippocampus, including CA1, CA3, and DG (Figure 3A). The aged mice showed a significant reduction in hippocampal neuronal cell numbers (Figures 3B–D, *p* < 0.0001). The administration of WHH2078 and WHH2080 significantly reversed the neuronal cell numbers in the area of CA1 (Figure 3B, *p* < 0.001), CA3 (Figure 3C, *p* < 0.0001), and DG (Figure 3D, *p* < 0.0001). The mice in the aged group had higher concentrations of neuroinflammatory cytokines in the hippocampus when compared to the young mice, including IL-1β (Figure 3E, *p* < 0.0001), IL-6 (Figure 3F, *p* < 0.0001) and TNFα (Figure 3G, *p* < 0.0001), which were attenuated following the administration of WHH2078 and WHH2080 (Figures 3E–G, *p* < 0.001). In addition, compared to the young mice, the hippocampal concentrations of neurochemical factors were significantly decreased in aged mice, including mBDNF (Figure 3H, *p* < 0.0001), 5-HT (Figure 3I, *p* < 0.0001), and 5-HTP (Figure 3J, *p* < 0.0001), which were ameliorated after administration of WHH2078 and WHH2080 (Figures 3H–J, *p* < 0.001).

### Effects of *Lactococcus lactis* WHH2078 and *Lactococcus cremoris* WHH2080 on circulating tryptophan metabolic profile in aged mice

**Figure 4:**
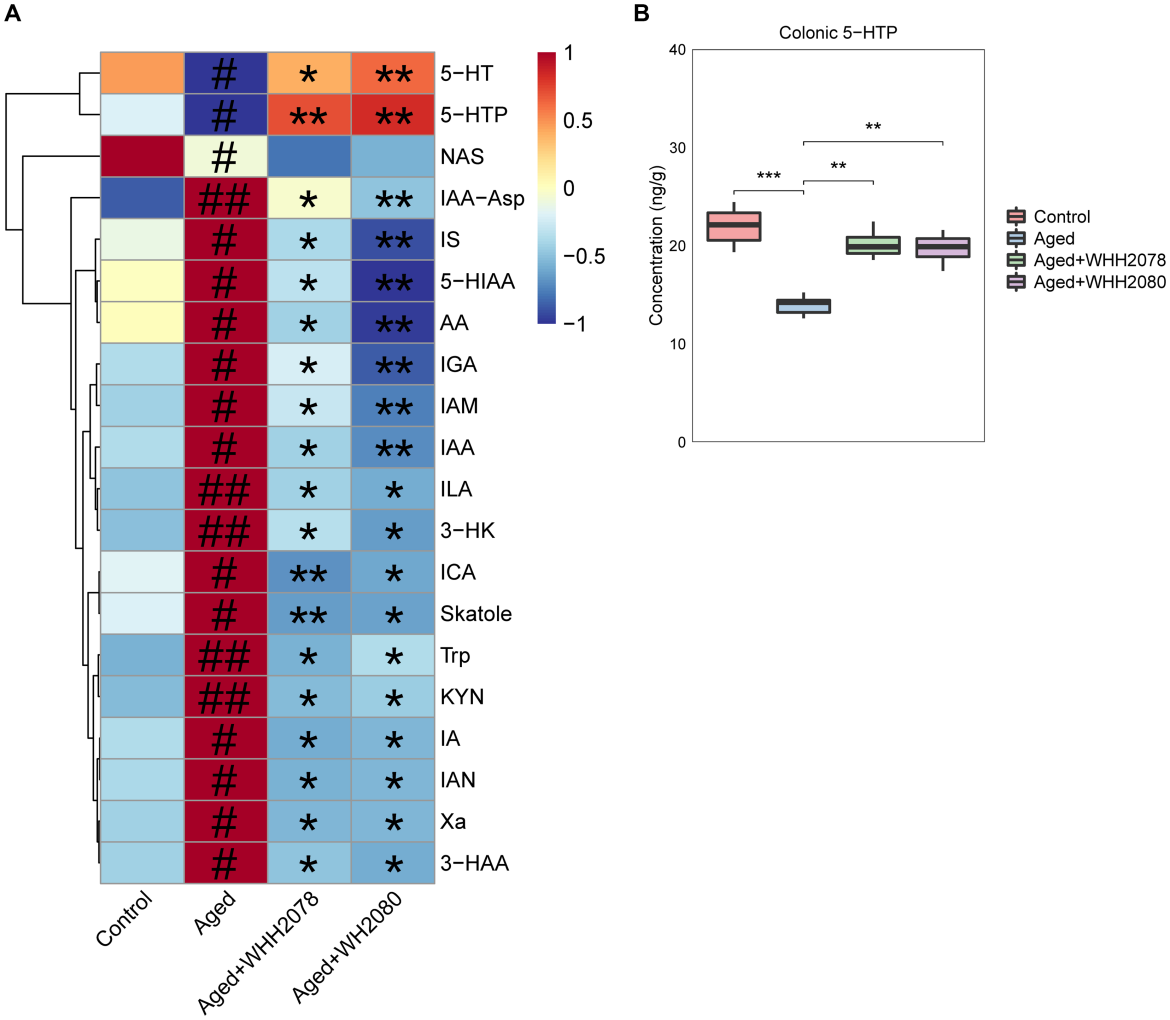
**(A)** The significantly altered tryptophan metabolites in the serum were revealed by the targeted metabolomics analysis (*n* = 8). The levels of tryptophan metabolites were normalized using the Z-score method, the red cell indicates a higher level, and the blue cell indicates a lower level. # *p* < 0.05, ## *p* < 0.01 vs. control group; * *p* < 0.05, ** *p* < 0.01 vs. aged group. **(B)** The levels of 5-HTP in the colon. Data are presented as medians ±95% CI, *n* = 8. * *p* < 0.05, ** *p* < 0.01, *** *p* < 0.001.

The changes in the profile of circulating tryptophan metabolites were disclosed through the tryptophan metabolism-targeted metabolomics (Figure 4A; Supplementary Table S3). Compared to the control group, the concentrations of 5-HT, 5-HTP, and NAS (Figure 4A, *p* < 0.05) were significantly decreased in the aged group. Conversely, the concentrations of IAA-Asp (*p* < 0.01), IS (*p* < 0.05), 5-HIAA (*p* < 0.05), AA (*p* < 0.05), IGA (*p* < 0.05), IAM (*p* < 0.05), IAA (*p* < 0.05), ILA (*p* < 0.01), 3-HK (*p* < 0.01), ICA (*p* < 0.05), Skatole (*p* < 0.05), Trp (*p* < 0.01), KYN (*p* < 0.01), IA (*p* < 0.05), IAN (*p* < 0.05), Xa (*p* < 0.05) and 3-HAA (*p* < 0.05) were significantly increased. Following the administration of WHH2078 and WHH2080, these alterations were significantly reversed (Figure 4A, *p* < 0.05), except for NAS (*p* < 0.05).

In addition, the aged mice displayed a significantly reduced amount of colonic 5-HTP (Figure 4B, *p* < 0.001) compared to the young mice, which were normalized by the treatment with WHH2078 and WHH2080 (Figure 4B, *p* < 0.01).

### Effects of *Lactococcus lactis* WHH2078 and *Lactococcus cremoris* WHH2080 on gut microbiome composition in aged mice

**Figure 5:**
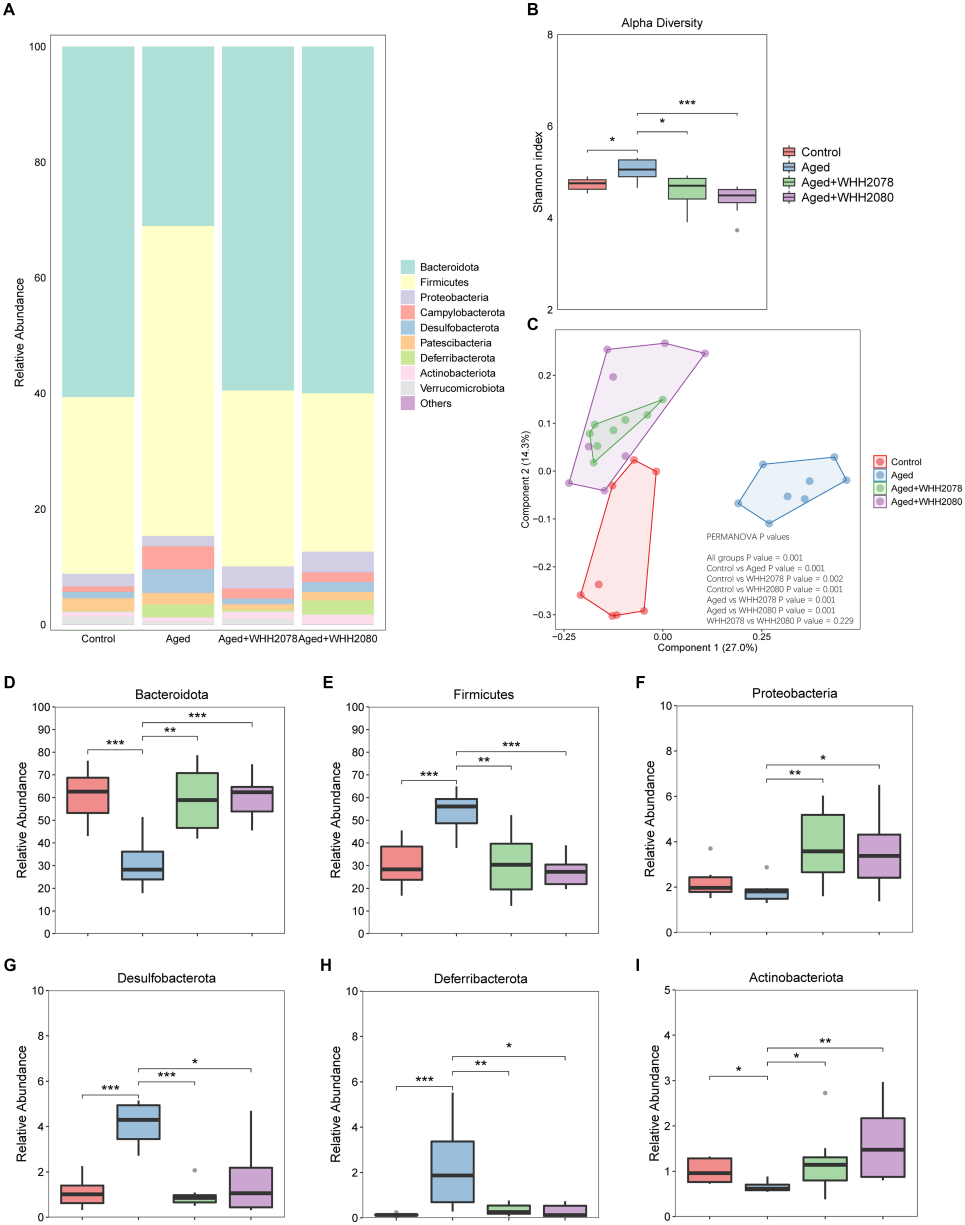
The changes in the gut microbiome composition in mice. **(A)** The composition of gut microbiome at the phylum level. **(B)** The Shannon index of the gut microbiome. **(C)** The Bray-Curtis distance-based PCoA. The PERMANOVA analysis was conducted at the feature level. *p* < 0.05 indicates the significant difference between groups. The abundances of phyla Bacteroidota **(D)**, Firmicutes **(E)**, Proteobacteria **(F)**, Desulfobacterota **(G)**, Deferribacterota **(H)**, and Actinobacteria **(I)**. Data are expressed as medians ±95% CI, *n* = 8. * *p* < 0.05, ** *p* < 0.01, *** *p* < 0.001.

The changes in the gut microbial community in aged mice following the treatments with WHH2078 and WHH2080 were revealed (Figure 5A). Compared to the control group, the Shannon index was increased in the aged group (Figure 5B, *p* < 0.05). The administration of WHH2078 and WHH2080 significantly normalized the increased alpha diversity in aged mice (Figure 5B, *p* < 0.05). Notably, considerable differences in the beta diversity were observed, which were supported by the evident group distinctions in the PCOA (Figure 5C, *p* = 0.001).

At the phylum level, compared to the young mice, the aged mice showed significantly decreased abundances of *Bacteroidota* (Figure 5D, *p* < 0.05) and *Actinobacteriota* (Figure 5I, *p* < 0.05), and significantly elevated abundances of *Firmicutes* (Figure 5E, *p* < 0.001), *Desulfobacterota* (Figure 5G, *p* < 0.001) and *Deferribacterota* (Figure 5H, *p* < 0.001). The dominant phyla changes in the aged mice were normalized by the administration of WHH2078 and WHH2080 (Figures 5D,E,G–I, *p* < 0.05). Additionally, the mice in the WHH2078 and WHH2080 groups showed higher abundances of *Proteobacteria* than those in the aged group (Figure 5F, *p* < 0.05).

**Figure 6:**
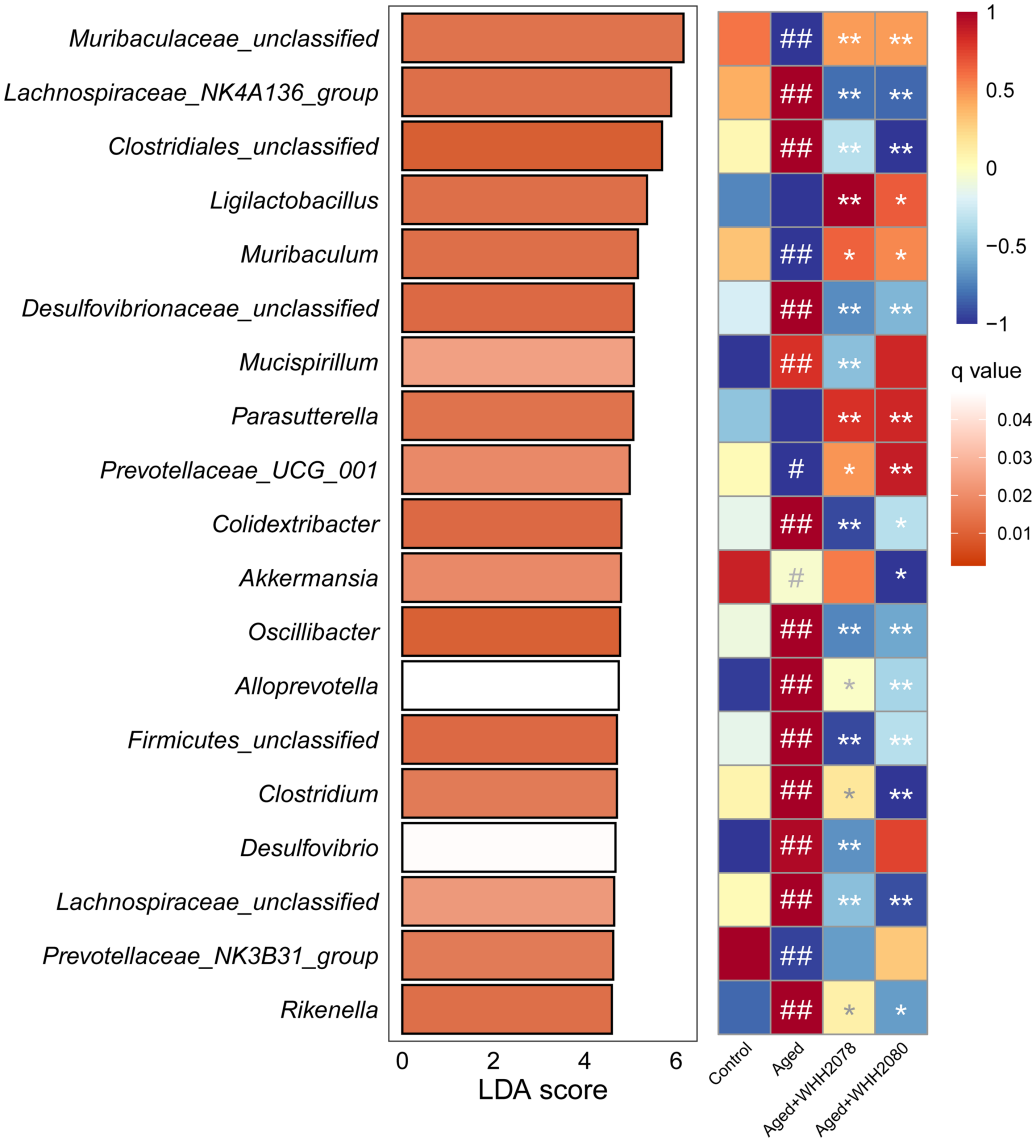
The significantly altered gut microbial genera in mice. The differential changes at the genus level were revealed using LefSe analysis with an LDA score > 4.0 and q value <0.05. Data were normalized into Z-scores (*n* = 8), the red cell indicates a higher abundance and the blue cell suggests a lower abundance. # *p* < 0.05, ## *p* < 0.01 vs. control group; * *p* < 0.05, ** *p* < 0.01 vs. aged group.

The LefSe analysis found the significantly altered genera at the genus level (LDA score 4.0 and q value 0.05) (Figure 6; Supplementary Table S4). Compared to the young mice, the aged mice displayed significantly higher abundances of *Lachnospiraceae_NK4A136_group*, *Clostridiales_unclassified*, *Desulfovibrionaceae_unclassified*, *Mucispirillum*, *Colidextribacter*, *Oscillibacter*, *Alloprevotella*, *Firmicutes_unclassified*, *Clostridium*, *Desulfovibrio*, *Lachnospiraceae_unclassified* and *Rikenella* (Figure 6, *p* < 0.01) and significantly lower abundances of *Muribaculaceae_unclassified* (*p* < 0.01), *Muribaculum* (*p* < 0.01), *Prevotellaceae_UCG_001* (*p* < 0.05), *Akkermansia* (*p* < 0.05) and *Prevotellaceae_NK3B31_group* (*p* < 0.01). Moreover, the alterations in the abundances of *Muribaculaceae_unclassified* (*p* < 0.01), *Lachnospiraceae_NK4A136_group* (*p* < 0.01), *Clostridiales_unclassified* (*p* < 0.01), *Muribaculum* (*p* < 0.05), *Desulfovibrionaceae_unclassified* (*p* < 0.01), *Prevotellaceae_UCG_001* (*p* < 0.05), *Colidextribacter* (*p* < 0.05), *Oscillibacter* (*p* < 0.01), *Alloprevotella* (*p* < 0.05), *Firmicutes_unclassified* (*p* < 0.01), *Clostridium* (*p* < 0.05), *Lachnospiraceae_unclassified* (*p* < 0.01) and *Rikenella* (*p* < 0.05) were reversed after the administration of WHH2078 and WHH2080. The elevated abundances of *Mucispirillum* and *Desulfovibrio* were reduced after the administration of WHH2078 (*p* < 0.01) and the reduced abundance of *Akkermansia* was reversed after WHH2080 treatment (*p* < 0.05). Compared to the aged group, the mice in the WHH2078 and WHH2080 groups showed significantly higher abundances of *Ligilactobacillus* (*p* < 0.05) and *Parasutterella* (*p* < 0.01). In addition, the abundance of *Lactococcus* was significantly increased after the administration of WHH2078 and WHH2080 compared to the aged group (Supplementary Figure S3, *p* < 0.05).

## Discussion

Aging always accompanies alterations in cognitive and mood functions (1). Probiotic strains are being increasingly recognized to alleviate cognitive and mood changes through the modulation of GBA function (12). Our previous study obtained two *Lactococcus* strains including *L. lactis* WHH2078 and *L. cremoris* WHH2080 with psychobiotic properties (15), exhibiting the most ability to enhance 5-HTP secretion among 19 *Lactococcus* strains (Supplementary Figure S4). In this study, the oral administration of WHH2078 and WHH2080 could mitigate cognitive impairment and mood disorders in aged mice. The changes in cognitive and mood behaviors, 5-HT metabolism, neuronal loss, neuroinflammation in the hippocampus, and gut microbiome composition were restored. Our findings suggest that *Lactococcus* strains, especially *L. lactis* WHH2078 and *L. cremoris* WHH2080 may serve as potential therapeutic agents to treat age-related cognitive impairment and mood disorders.

In the current study, the 12-month-old mice exhibited evident cognitive declines, as evidenced by the hippocampal neuronal loss and learning and non-spatial/spatial memory impairments revealed by cognitive behavioral tests, which are consistent with previous reports (23, 24). Although the aging process is complex, emerging evidence indicates that cognitive declines in aged people are accompanied by mood disorders, including depression and anxiety (1). Consistently, aged mice in this study also exhibited depressive-and anxiety-like behaviors, as evidenced by the fewer FST immobility times and time spent in the central area in OFT. Therefore, our findings indicate the 12-month-old mouse was a suitable animal model for evaluating the psychobiotic effects of candidate strains.

Numerous studies report probiotic strains with psychobiotic properties (12). Certain strains typically strain within *Lactiplantibacillus plantarum* (8, 9), *Lactobacillus helveticus* (10), and *Bifidobacterium longum* (11), are well-recognized for alleviating both cognitive alterations and mood disorders. Strains belonging to *Lactococcus* including *L. lactis* (15) and *L. cremoris* (16) have been reported to improve depressive and anxiety symptoms. In the present study, the abnormalities in the age-related cognitive and mood functions were reversed following the treatment with WHH2078 and WHH2080, highlighting the psychobiotic abilities within the *Lactococcus* strains.

The 5-HT metabolism is crucial to the GBA (3). Disturbances in the 5-HT metabolism are frequently linked to the emergence of neuropsychiatric diseases, such as depression and cognitive decline (25). Gut microbiota exerts an important role in the modulation of 5-HT metabolism through directly synthesizing 5-HT or promoting the production of 5-HT in the intestinal enterochromaffin cells (EEC) (17). Nevertheless, the gut-derived 5-HT cannot affect the central 5-HT pool directly due to the existence of BBB (17). 5-HT is mainly synthesized from tryptophan, one of the essential amino acids, and 5-HTP was an important intermediate in the biosynthesis of 5-HT, which can raise 5-HT levels in the brain by crossing BBB, thereby having positive effects on CNS functions (26). Supplementation of probiotics such as *Bifidobacterium* [such as *B. longum* (27) and *B. breve* (28)] and *Lactobacillus* [such as *L. helveticus* (20)] with the gut 5-HTP-promoting ability can restore the central 5-HT pool via the GBA route. Therefore, the *in vitro* EEC cell model was developed to screen the probiotic strains with 5-HTP-stimulating ability (18). Two best strains WHH2078 and WHH2080 from nineteen *Lactococcus* strains that could promote gut 5-HTP secretion were obtained in our previous study (15). After the treatments of WHH2078 and WHH2080, the decreased levels of hippocampal 5-HT and 5-HTP were restored. As evidenced by the increased circulating levels of 5-HT and 5-HTP, as well as the elevated production of 5-HTP in the colon, the present findings indicate the psychobiotic effects of WHH2078 and WHH2080 may be partly through a 5-HTP-promoting manner.

The metabolomics unveiled the changes in the circulating tryptophan metabolism following the administration of two *Lactococcus* strains. Consistent with a previous study (20), the decreased 5-HT synthesis pathway, the enhanced kynurenine (KYN) metabolism, and microbial tryptophan metabolism were observed in aged mice. The accumulations of key metabolites in the KYN metabolism, such as 3-HAA, 3-HK, and Xa are regarded as neurotoxins, which are detrimental to cognition and mood function (29). Meanwhile, the microbial tryptophan utilization was also elevated, as evidenced by the increased levels of indolic compounds, such as ILA, IAA, ICA, IGA, and skatole, which are important mediators in the host-microbial interaction (30). Interestingly, the disturbances in the circulating tryptophan metabolism were reversed after the treatments with WHH2078 and WHH2080, which may be due to the ability of these two strains to promote the colonic 5-HTP secretion, thereby increasing the peripheral 5-HT pool and normalizing the circulating tryptophan metabolism.

The existence of GAB is well-recognized, and gut dysbiosis is often associated with the development of CNS dysfunctions (13). During the process of aging, the gut microbiome composition changes over time (31). In the current study, the gut microbiome composition in aged mice was different from that in young mice. Regarding the top dominant phyla, the increase in *Firmicutes* and the decrease in *Bacteroidota* and *Actinobacteriota* were observed in aged mice, which was consistent with previous studies (20, 32). However, it is worth mentioning that there is currently no consensus on the changes in the bacterial dominant phyla in mice associated with aging, due to various factors such as genetic background, age, and diet. Several studies inconsistent with our results have also been reported (33, 34). In this study, supplementation of *Lactococcus* strains WHH2078 and WHH2080 restored these changes. In addition, some phyla such as *Desulfobacterota* and *Deferribacterota* were increased in aged mice. However, bacteria within the phyla *Desulfobacterota* and *Deferribacterota* are often responsible for inducing the host’s systemic inflammation (35). WHH2078 and WHH2080 reduced the abundances of *Desulfobacterota* and *Deferribacterota*. These findings highlight the modulatory role of these two *Lactococcus* strains in the gut microbiome composition of mice associated with aging, and the effects may have a potentially beneficial influence on host health.

The genus-level changes were unveiled by LefSe analysis, and the most changed genus in aged mice belongs to the family *Muribaculaceae*, which is closely associated with the development of neurocognitive and mental health disorders (36). Consistent with a previous study (20), the predominant genus *Muribaculum* was also decreased in aged mice, and *Muribaculum* acts as a biomarker in cognitive impairment, linking to cognitive decline (37). The administration of WHH2078 and WHH2080 can reverse these changes. In addition to the increase in the abundance of *Lactococcus* (Supplementary Figure S1), these two strains increased *Ligilactobaccilus* and reduced *Oscillibacter*, which are associated with the pathophysiology of cognitive and mood functions (38, 39). The current results disclosed the administration of WHH2078 and WHH2080 could reverse the changes in the microbial genera, indicating that two *Lactococcus* strain may exert their psychobiotic effects partly by reshaping these targeted genera.

Some limitations should be explained. Firstly, since the GAB is bidirectional (13), the conclusion of the current study cannot simply draw that two *Lactococcus* strains mediated brain function by modulating the gut microbes, the causal role of WHH2078 and WHH2080 in the underlying mechanisms will be investigated further. Secondly, due to the differences in the environmental and host factors, such as genetic background, gender, diets, and gut microbial structure (40), the effectiveness in animal models is not equal to that in humans, future clinical validation must be performed.

## Conclusion

**Figure 7:**
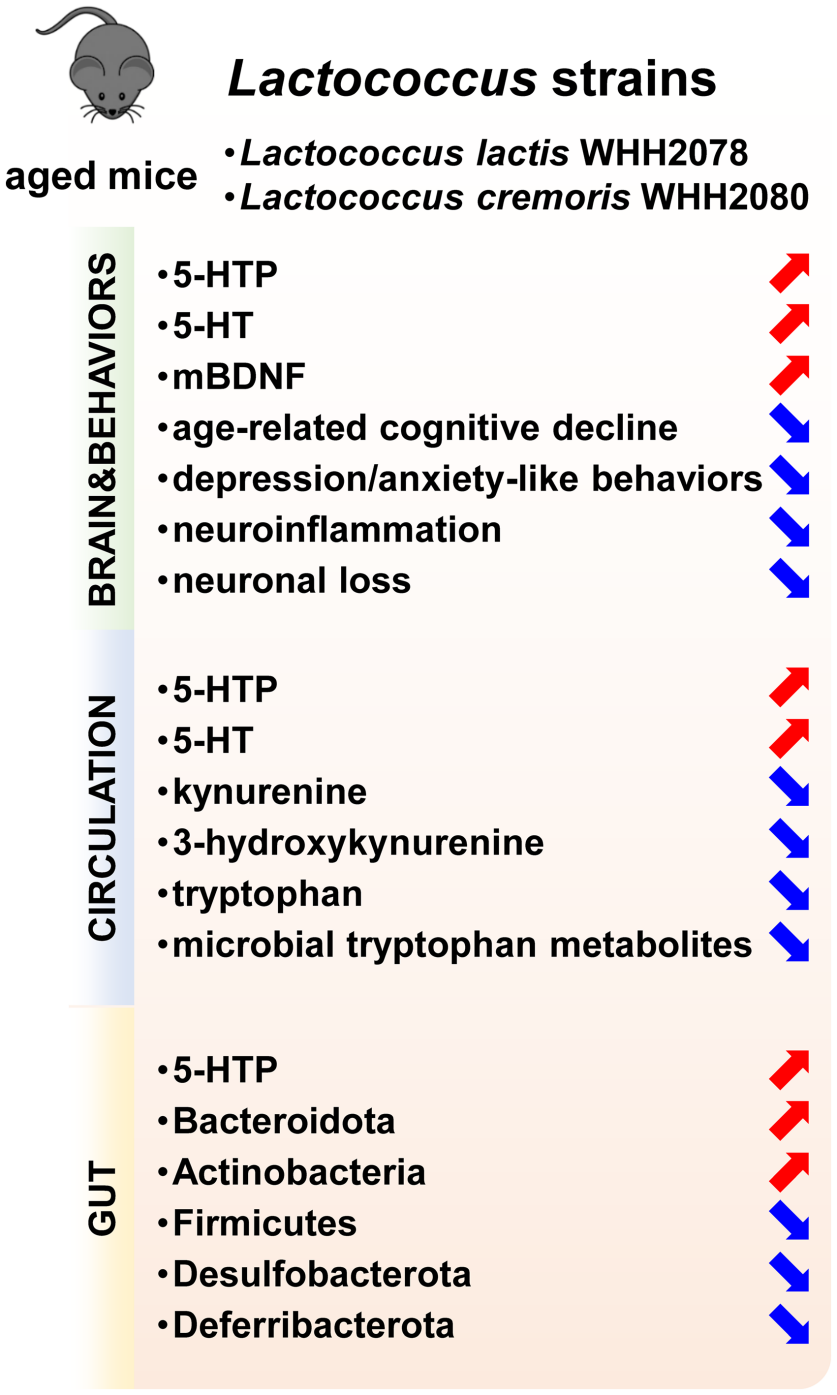
The psychobiotic effects of *Lactococcus* strains including *L. lactis* WHH2078 and *L. cremoris* WHH2080 on aged mice. Increased parameters were labeled with red arrows, and decreased parameters were labeled with blue arrows.

In conclusion, the current investigation demonstrated that the oral administration of *Lactococcus* strains *L. lactis* WHH2078 and *L. cremoris* WHH2080 could alleviate the cognitive and mood alterations in aged mice, which was linked to the strain-mediated modulation of gut microbiome composition and 5-HT metabolism (Figure 7). The current findings provide evidence supporting the potential role of *Lactococcus* strains with intestinal 5-HTP-promoting abilities as novel therapeutic strategies for age-related cognitive and mood disorders.

## Data availability statement

The datasets presented in this study can be found in online repositories. The names of the repository/repositories and accession number(s) can be found at: https://www.ncbi.nlm.nih.gov/, PRJNA1021314.

## Ethics statement

The animal study was approved by the Animal Care and Use Committee of Hangzhou Wahaha Technology Co., Ltd. The study was conducted in accordance with the local legislation and institutional requirements.

## Author contributions

KG: Conceptualization, Formal analysis, Investigation, Methodology, Visualization, Writing – original draft, Writing – review & editing. CC: Data curation, Investigation, Methodology, Writing – original draft. ZZ: Investigation, Methodology, Validation, Writing – original draft. QF: Methodology, Resources, Validation, Writing – review & editing. HW: Supervision, Writing – review & editing. YL: Conceptualization, Resources, Supervision, Writing – review & editing. SC: Funding acquisition, Resources, Supervision, Writing – review & editing.
