# Supplementary material for: Lactococcus strains with psychobiotic properties improve cognitive and mood alterations in aged mice
Source: Front Nutr. 2024 Aug 1;11:1439094. doi: 10.3389/fnut.2024.1439094 (PMC11324604; doi:10.3389/fnut.2024.1439094)
Supplement: Supplementary file 1 [file Data_Sheet_1.docx]

**Supplementary Information**

***Lactococcus* strains with psychobiotic properties improve cognitive and mood alterations in aged mice**

Kan Gao^a, b, c^, Cailing Chen^a, b, c^, Zhiyao Zheng^a, b, c^, Qiuling Fan^a, b, c^, Haifeng Wang^d^, Yanjun Li^a, b, c^, Su Chen^a, b, c *^

^a^ *Research and Development Department, Hangzhou Wahaha Group Co., Ltd, Hangzhou 310018, P.R. China*

*^b^ Hangzhou Wahaha Technology Co., Ltd, Hangzhou 310018, P.R. China*

^c^ *Key Laboratory of Food And Biological Engineering of Zhejiang Province, Hangzhou 310018, P.R. China*

^d^ *College of Animal Science, MOE Key Laboratory of Molecular Animal Nutrition, Zhejiang University, Hangzhou 310058, P.R. China*

* Corresponding Author:

Su Chen, Ph.D., Associate Professor

Email Address: chensu@wahaha.com.cn

Tel: 0086-13758148182

Fax: 0086-571-86796090

**SUPPLEMENTARY TABLES**

**Table S1.** The accuracy and precision of the ELISA assays in this study

| **Items in the hippocampus** | **r^2^** | **Accuracy (%)^1^** | **Precision (%)^2^** |
| --- | --- | --- | --- |
| 5-HT | 0.9998 | 5.06 | 3.21 |
| mature BDNF | 0.9999 | 5.14 | 3.04 |
| IL-1β | 0.9999 | 5.18 | 2.96 |
| IL-6 | 0.9999 | 5.65 | 2.98 |
| TNF-α | 0.9998 | 5.66 | 3.22 |

^1^ The accuracy (the closeness of an experimental value to the true value) of the method, as determined with known amounts of neurochemical factors standards and expressed as the relative errors [(measurement value – true value)/true value × 100%)]. A value below 10 % indicates the method is highly accurate.

^2^ The precision (agreement between replicate measurements) of the method, as evaluated by the relative deviation (mean of absolute deviation/mean of replicate measurements × 100%). A value below 5 % indicates the method is highly precise.

**Table S2.** The compounds of tryptophan metabolites used in metabolomics analysis

| **Metabolites** | **Abbreviation** | **CAS Number** | **Formula** |
| --- | --- | --- | --- |
| 3-Hydroxyanthranilic acid | 3-HAA | 548-93-6 | C_7_H_7_NO_3_ |
| 3-Hydroxykynurenine | 3-HK | 2147-61-7 | C_10_H_12_N_2_O_4_ |
| 5-Hydroxyindoleacetic acid | 5-HIAA | 54-16-0 | C_10_H_9_NO_3_ |
| Serotonin | 5-HT | 50-67-9 | C_10_H_12_N_2_O |
| 5-Hydroxytryptophol | 5-HTOL | 154-02-9 | C_10_H_11_NO_2_ |
| L-5-Hydroxytryptophan | 5-HTP | 4350-09-8 | C_11_H_12_N_2_O_3_ |
| 5-Methoxy-3-indoleacetic acid | 5-Me-IAA | 3471-31-6 | C_11_H_11_NO_3_ |
| Anthranilic acid | AA | 118-92-3 | C_7_H_7_NO_2_ |
| Indole acrylic acid | IA | 29953-71-7 | C_11_H_9_NO_2_ |
| Indole-3-acetic acid | IAA | 87-51-4 | C_10_H_9_NO_2_ |
| Indole-3-acetamide | IAM | 879-37-8 | C_10_H_10_N_2_O |
| Indole-3-carboxaldehyde | ICA | 487-89-8 | C_9_H_7_NO |
| Indole ethanol/tryptophol | IE | 526-55-6 | C_10_H_11_NO |
| 3-Indoleglyoxylic acid | IGA | 1477-49-2 | C_10_H_7_NO_3_ |
| Indolelactic acid | ILA | 1821-52-9 | C_11_H_11_NO_3_ |
| Indican | Indican | 487-60-5 | C_14_H_17_NO_6_ |
| Indole | Indole | 120-72-9 | C_8_H_7_N |
| 3-Indolepropionic acid | IPA | 830-96-6 | C_11_H_11_NO_2_ |
| Indoxylsulfate | IS | 2642-37-7 | C_8_H_7_NO_4_S |
| Kynurenine | KYN | 343-65-7 | C_10_H_12_N_2_O_3_ |
| Kynurenic acid | KYNA | 492-27-3 | C_10_H_7_NO_3_ |
| Melatonin | Melatonin | 73-31-4 | C_13_H_16_N_2_O_2_ |
| Nicotinic acid | NA | 59-67-6 | C_6_H_5_NO_2_ |
| N-Acetyl-5-hydroxytryptamine | NAS | 1210-83-9 | C_12_H_14_N_2_O_2_ |
| Skatole | Skatole | 83-34-1 | C_9_H_9_N |
| L-Tryptophan | Trp | 73-22-3 | C_11_H_12_N_2_O_2_ |
| Tryptamine | Tryptamine | 61-54-1 | C_10_H_12_N_2_ |
| Xanthurenic acid | Xa | 59-00-7 | C_10_H_7_NO_4_ |
| Indole-3-acetonitrile | IAN | 771-51-7 | C_10_H_8_N_2_ |
| Indole-3-acetyl-alanine | IAA-Ala | 57105-39-2 | C_13_H_14_N_2_O_3_ |
| Indole-3-acetyl-aspartate | IAA-Asp | 2456-73-7 | C_14_H_14_N_2_O_5_ |

**Table S3.** The concentrations of tryptophan metabolites

| **Items (nmol/L)** | **Group^1^** | | | | **P values** |
| --- | --- | --- | --- | --- | --- |
|  | **Control** | **Aged** | **Aged+WHH2078** | **Aged+WH2080** |  |
| 3-HAA | 20.03±2.25 ^b^ | 44.48±25.35 ^a^ | 19.29±2.03 ^b^ | 18.10±1.18 ^b^ | ＜0.001 |
| 3-HK | 143.48±10.23 ^b^ | 347.66±32.20 ^a^ | 160.04±15.83 ^b^ | 128.05±10.11 ^b^ | ＜0.001 |
| 5-HIAA | 709.80±35.89 ^b^ | 973.18±38.30 ^a^ | 648.56±24.16 ^b^ | 510.15±19.15 ^c^ | ＜0.001 |
| 5-HT | 15326.64±495.48 ^a^ | 13352.50±173.35 ^b^ | 15261.39±419.59 ^a^ | 15503.37±282.77 ^a^ | ＜0.001 |
| 5-HTOL | 3.62±0.33 | 4.18±0.35 | 4.29±0.54 | 3.29±0.19 | 0.213 |
| 5-HTP | 196.22±7.61 ^b^ | 163.34±7.56 ^c^ | 222.15±8.85 ^a^ | 226.08±6.40 ^a^ | ＜0.001 |
| 5-Me-IAA | 0.28±0.04 | 0.24±0.04 | 0.26±0.03 | 0.23±0.03 | 0.842 |
| AA | 51.70±5.05 ^b^ | 77.07±5.82 ^a^ | 43.42±2.17 ^bc^ | 33.27±1.65 ^c^ | ＜0.001 |
| IA | 79.98±7.42 ^b^ | 157.94±15.79 ^a^ | 70.67±7.36 ^b^ | 72.14±3.45 ^b^ | ＜0.001 |
| IAA | 632.97±33.07 ^b^ | 1064.63±75.06 ^a^ | 619.00±34.93 ^b^ | 549.10±27.01 ^b^ | ＜0.001 |
| IAA-Asp | 0.30±0.14 ^c^ | 2.37±0.21 ^a^ | 1.05±0.32 ^b^ | 0.65±0.21 ^bc^ | ＜0.001 |
| IAM | 0.30±0.04 ^b^ | 0.56±0.06 ^a^ | 0.32±0.02 ^b^ | 0.26±0.02 ^b^ | ＜0.001 |
| IAN | 0.32±0.04 ^b^ | 0.52±0.05 ^a^ | 0.31±0.02 ^b^ | 0.31±0.02 ^b^ | ＜0.001 |
| ICA | 257.15±22.22 ^b^ | 405.58±29.46 ^a^ | 211.26±12.92 ^b^ | 219.35±25.61 ^b^ | ＜0.001 |
| IE | 1.19±0.22 | 1.35±0.05 | 1.02±0.08 | 1.05±0.11 | 0.375 |
| IGA | 52.60±5.40 ^bc^ | 91.46±6.96 ^a^ | 55.60±1.89 ^b^ | 41.63±2.71 ^c^ | ＜0.001 |
| ILA | 1785.30±70.60 ^b^ | 2717.22±200.85 ^a^ | 1813.33±115.38 ^b^ | 1743.97±106.55 ^b^ | ＜0.001 |
| Indican | 116.53±14.87 | 145.63±15.77 | 111.69±8.31 | 125.94±7.45 | 0.232 |
| IPA | 3422.81±321.65 | 3187.78±219.01 | 2802.07±300.97 | 2997.54±230.13 | 0.428 |
| IS | 17725.43±1348.25 ^b^ | 26074.10±1107.71 ^a^ | 16352.26±1218.39 ^bc^ | 13466.24±1029.70 ^c^ | ＜0.001 |
| KYN | 853.26±26.91 ^b^ | 1477.31±94.95 ^a^ | 850.40±52.91 ^b^ | 880.44±41.03 ^b^ | ＜0.001 |
| KYNA | 128.83±16.27 | 174.17±23.01 | 136.04±27.58 | 126.61±18.15 | 0.387 |
| NA | 254.80±254.23 | 0.62±0.62 | 1.90±0.98 | 26.41±15.46 | 0.437 |
| NAS | 22.50±2.00 ^a^ | 15.93±0.80 ^b^ | 12.93±0.88 ^b^ | 13.87±0.59 ^b^ | ＜0.001 |
| Skatole | 8.05±1.16 ^b^ | 19.66±4.35 ^a^ | 4.91±0.72 ^b^ | 4.96±0.72 ^b^ | ＜0.001 |
| Trp | 107397.60±2633.05 ^b^ | 145081.16±5391.19 ^a^ | 107119.72±3425.48 ^b^ | 110925.20±4041.22 ^b^ | ＜0.001 |
| Xa | 300.88±7.46 ^b^ | 459.49±13.34 ^a^ | 290.71±10.17 ^b^ | 290.30±10.86 ^b^ | ＜0.001 |
| Indole | n.d.^*^ | n.d. | n.d. | n.d. | / |
| IAA-Ala | n.d. | n.d. | n.d. | n.d. | / |
| Melatonin | n.d. | n.d. | n.d. | n.d. | / |
| Tryptamine | n.d. | n.d. | n.d. | n.d. | / |

^1^ Data are presented as means ± SEM. The different letters indicate significant differences (*P*<0.05).

^*^n.d., not detected.

**Table S4.** The significantly changed genera revealed by the LefSe analysis (LDA score＞4.5, q value＜0.05)

| **Genera** | **LDA score** | **q value^1^** | **Group^2^** | | | | ***P* values^3^** |
| --- | --- | --- | --- | --- | --- | --- | --- |
|  |  |  | **Control** | **Aged** | **Aged+WHH2078** | **Aged+WHH2080** |  |
| *Muribaculaceae_unclassified* | 6.16 | 0.006 | 51.00±3.43^a^ | 21.90±3.03^b^ | 48.50±4.69^a^ | 48.44±3.27^a^ | <0.001 |
| *Lachnospiraceae_NK4A136_group* | 5.89 | 0.005 | 9.13±2.93^b^ | 18.66±2.24^a^ | 3.28±0.77^c^ | 3.20±0.99^c^ | <0.001 |
| *Clostridiales_unclassified* | 5.69 | 0.002 | 2.23±0.51^b^ | 10.47±2.24^a^ | 1.36±0.37^b^ | 0.58±0.13^b^ | <0.001 |
| *Ligilactobacillus* | 5.36 | 0.005 | 1.13±0.23^b^ | 0.90±0.23^b^ | 5.52±2.13^a^ | 3.99±1.07^a^ | 0.001 |
| *Muribaculum* | 5.16 | 0.005 | 3.79±1.07^a^ | 1.54±0.19^b^ | 4.42±0.51^a^ | 4.17±0.47^a^ | <0.001 |
| *Desulfovibrionaceae_unclassified* | 5.07 | 0.004 | 0.72±0.18^b^ | 2.82±0.31^a^ | 0.48±0.11^b^ | 0.55±0.20^b^ | <0.001 |
| *Mucispirillum* | 5.07 | 0.019 | 0.13±0.02^b^ | 2.33±0.70^a^ | 0.35±0.09^b^ | 2.48±1.65^a^ | 0.008 |
| *Parasutterella* | 5.06 | 0.006 | 1.29±0.24^b^ | 0.82±0.21^b^ | 3.05±0.56^a^ | 3.14±0.60^a^ | 0.001 |
| *Prevotellaceae_UCG_001* | 4.98 | 0.012 | 1.21±0.31^b^ | 0.41±0.12^c^ | 1.71±0.46^ab^ | 2.33±0.77^a^ | 0.004 |
| *Colidextribacter* | 4.80 | 0.004 | 0.52±0.08^b^ | 1.55±0.21^a^ | 0.29±0.07^b^ | 0.45±0.14^b^ | <0.001 |
| *Akkermansia* | 4.79 | 0.012 | 1.33±0.39^a^ | 0.48±0.22^b^ | 0.95±0.63^ab^ | 0.09±0.02^c^ | 0.004 |
| *Oscillibacter* | 4.77 | 0.002 | 0.34±0.04^b^ | 1.38±0.20^a^ | 0.20±0.05^b^ | 0.22±0.10^b^ | <0.001 |
| *Alloprevotella* | 4.74 | 0.047 | 0.44±0.14^b^ | 1.55±0.34^a^ | 0.73±0.21^b^ | 0.60±0.17^b^ | 0.009 |
| *Firmicutes_unclassified* | 4.70 | 0.004 | 0.55±0.06^b^ | 1.36±0.15^a^ | 0.35±0.09^b^ | 0.49±0.12^b^ | <0.001 |
| *Clostridium* | 4.70 | 0.008 | 0.47±0.12^b^ | 1.13±0.22^a^ | 0.50±0.31^b^ | 0.14±0.04^b^ | 0.002 |
| *Desulfovibrio* | 4.67 | 0.046 | 0.39±0.10^b^ | 1.33±0.23^a^ | 0.48±0.10^b^ | 1.17±0.42^a^ | 0.023 |
| *Lachnospiraceae_unclassified* | 4.64 | 0.017 | 0.56±0.10^b^ | 1.21±0.22^a^ | 0.42±0.06^b^ | 0.33±0.07^b^ | <0.001 |
| *Prevotellaceae_NK3B31_group* | 4.62 | 0.008 | 0.91±0.25^a^ | 0.07±0.05^b^ | 0.10±0.04^b^ | 0.30±0.16^b^ | 0.002 |
| *Rikenella* | 4.59 | 0.005 | 0.28±0.06^b^ | 0.87±0.16^a^ | 0.47±0.09^b^ | 0.44±0.11^b^ | 0.005 |

^1^ q values were calculated based on the *P* values from the LefSe analysis with FDR corrections.

^2^ Data are presented with means ± SEM. The different letters indicate significant differences (*P*<0.05).

^3^ The *P* values were calculated from the One-way ANOVA analysis or Kruskal-Wallis test.

**SUPPLEMENTARY FIGURES**

**
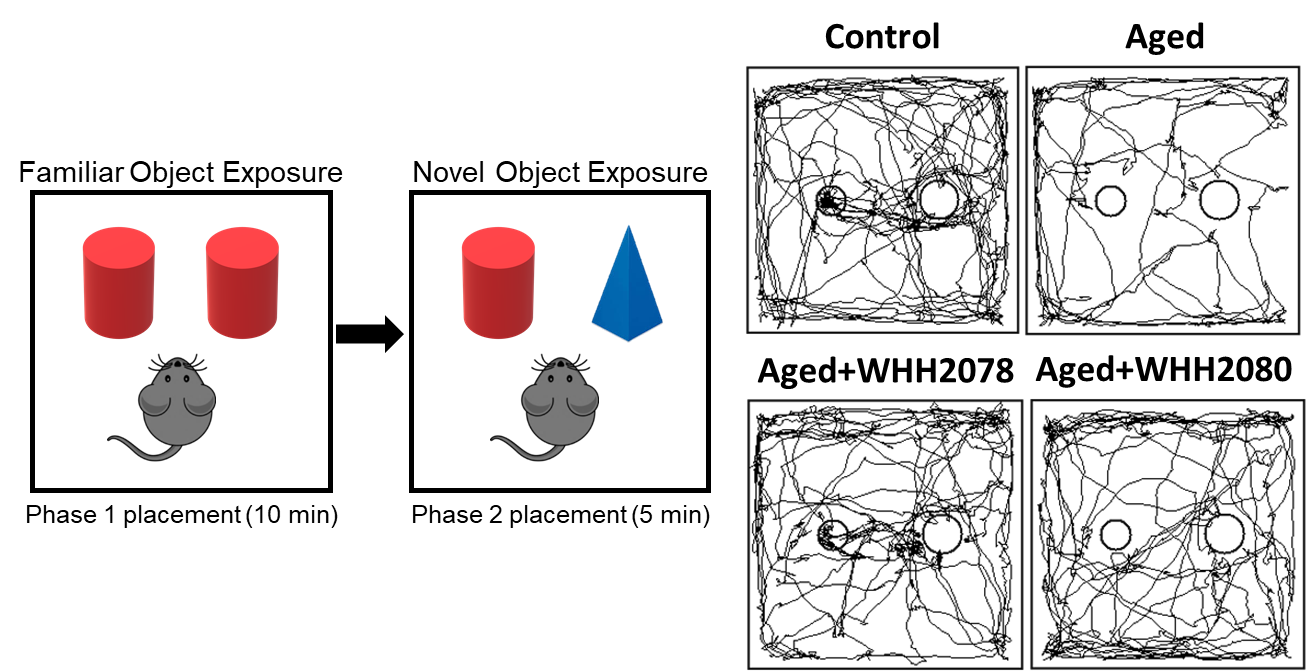
**

**Figure S1.** Tracking movement in the novel object recognition test.

**
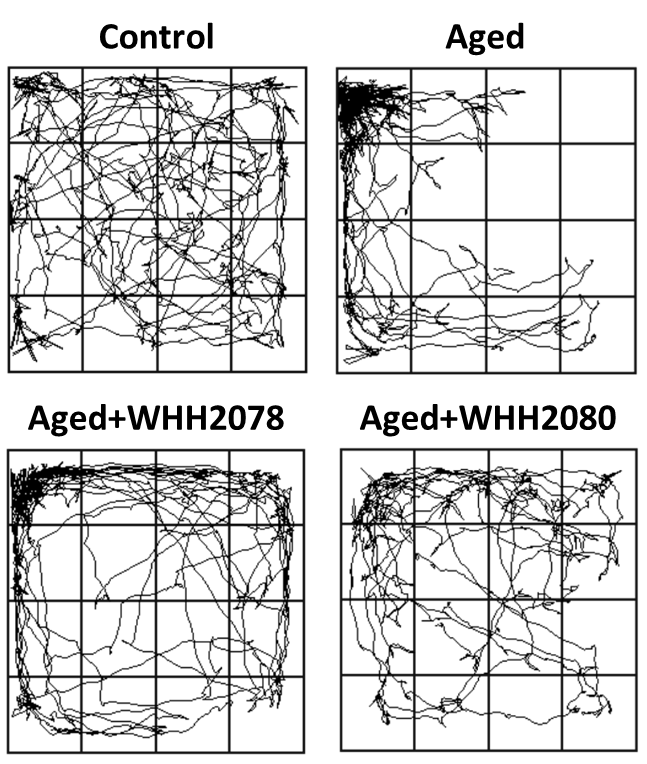
**

**Figure S2.** Tracking movement in the open field test.


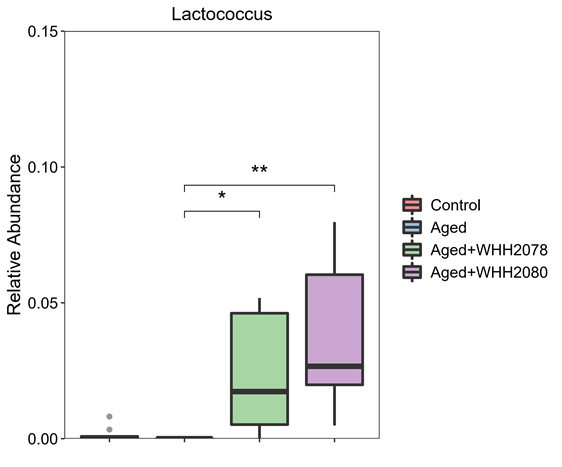


**Figure S3.** The changes in the abundance of *Lactococcus*.

Data are presented as medians ± 95% CI (n=8). * *P* < 0.05, ** *P* < 0.01.


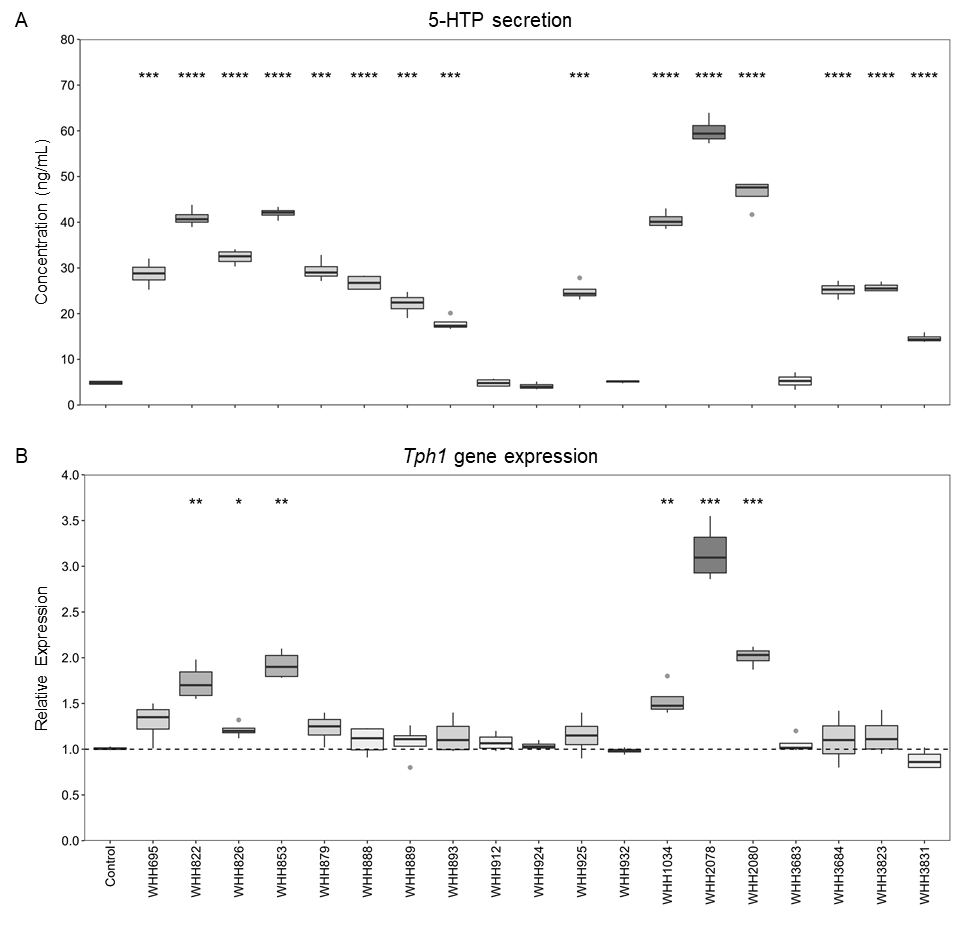


**Fig. S4** Screening the ability of *Lactococcus lactis* strains in RIN14B cells for promoting 5-HTP level (*published in Gao et al., 2022*). (A) The levels of 5-HTP. (B) The *Tph1* gene expressions. Data are expressed as the medians ± 95% CI, with 4 replicates included in each analysis. *Statistically significant differences between control and *Lactococcus lactis* strains. * *P* < 0.05, ** *P* < 0.01, *** *P* < 0.001, **** *P* < 0.0001.
